# Proteomic and functional analysis of NCS-1 binding proteins reveals novel signaling pathways required for inner ear development in zebrafish

**DOI:** 10.1186/1471-2202-10-27

**Published:** 2009-03-25

**Authors:** Jessica A Petko, Nadine Kabbani, Colleen Frey, Matthew Woll, Katharine Hickey, Michael Craig, Victor A Canfield, Robert Levenson

**Affiliations:** 1 Department of Pharmacology, Penn State College of Medicine, 500 University Drive, Hershey PA 17033, USA; 2 Department of Biology, Gettysburg College, 300 North Washington Street, Gettysburg PA 17325, USA; 3 Department of Molecular Neuroscience, Krasnow Institute for Advanced Study, George Mason University, 4400 University Drive, Fairfax VA 22030, USA

## Abstract

**Background:**

The semicircular canals, a subdivision of the vestibular system of the vertebrate inner ear, function as sensors of angular acceleration. Little is currently known, however, regarding the underlying molecular mechanisms that govern the development of this intricate structure. Zebrafish represent a particularly tractable model system for the study of inner ear development. This is because the ear can be easily visualized during early embryogenesis, and both forward and reverse genetic techniques are available that can be applied to the discovery of novel genes that contribute to proper ear development. We have previously shown that in zebrafish, the calcium sensing molecule neuronal calcium sensor-1 (NCS-1) is required for semicircular canal formation. The function of NCS-1 in regulating semicircular canal formation has not yet been elucidated.

**Results:**

We initiated a multistep functional proteomic strategy to identify neuronal calcium sensor-1 (NCS-1) binding partners (NBPs) that contribute to inner ear development in zebrafish. By performing a Y2H screen in combination with literature and database searches, we identified 10 human NBPs. BLAST searches of the zebrafish EST and genomic databases allowed us to clone zebrafish orthologs of each of the human NBPs. By investigating the expression profiles of zebrafish NBP mRNAs, we identified seven that were expressed in the developing inner ear and overlapped with the *ncs-1a *expression profile. GST pulldown experiments confirmed that selected NBPs interacted with NCS-1, while morpholino-mediated knockdown experiments demonstrated an essential role for *arf1*, *pi4kβ, dan*, and *pink1 *in semicircular canal formation.

**Conclusion:**

Based on their functional profiles, the hypothesis is presented that Ncs-1a/Pi4kβ/Arf1 form a signaling pathway that regulates secretion of molecular components, including Dan and Bmp4, that are required for development of the vestibular apparatus. A second set of NBPs, consisting of Pink1, Hint2, and Slc25a25, are destined for localization in mitochondria. Our findings reveal a novel signalling pathway involved in development of the semicircular canal system, and suggest a previously unrecognized role for NCS-1 in mitochondrial function via its association with several mitochondrial proteins.

## Background

The vestibular system of the vertebrate inner ear is a highly complex set of structures that relays information about motion and spatial orientation to the brain. The structural and functional properties of the vestibular apparatus are highly conserved among all vertebrate species. The semicircular canals, one subdivision of the vestibular system, function as sensors of angular acceleration. These structures enhance survival by implementing postural and visual stabilization during motion in a three-dimensional environment [1-3].

Zebrafish has emerged as an important model system for the study of ear development. Zebrafish represent an especially attractive system for analyzing otogenesis, owing to the fact that the ear can be easily visualized during early embryogenesis, and that many mutations in ear development have been described in zebrafish that appear to resemble those that cause human hearing and balance disorders (reviewed in [4]). In zebrafish, as well as other vertebrates, the semicircular canal system consists of three non-sensory components, the anterior, posterior, and lateral canal ducts (reviewed in [5]) which are positioned in three different planes. Sensory regions lie at the end of each canal and detect differential flow of fluid through the non-sensory structures during angular rotation of the head.

The formation of the semicircular canals in zebrafish begins at 45 hpf with the development of three epithelial outpocketings: one anterior protrusion, one posterior protrusion, and one lateral protrusion [6]. At 60 hpf, the lateral protrusion bifurcates into an anterior branch and a posterior branch that fuse with the anterior and posterior protrusions, respectively [7]. Shortly after the formation of the horizontal hub, a ventral protrusion develops at the bottom of the vesicle and fuses with the lateral protrusion. Finally, a thin septum arises from the dorsal wall forming a dorsolateral partition. At 72 hpf, the four hubs are completely formed and appear as a cross shaped structure that spans the lumen of the otic vesicle. Little is currently known, however, regarding the underlying molecular mechanisms that govern the development of this intricate structure.

Mutagenesis screens in zebrafish have uncovered several types of mutants with semicircular canal defects [8-10]. For example, a mutation in the *jekyll *gene leads to interrupted ear columns (epithelial pillars which form the hubs of the semicircular canals) and malformations of pharyngeal cartilage [11]. The *jekyll *gene encodes Ugdh, an enzyme required for the production of several components of the extracellular matrix including hyaluronic acid [12]. Localized secretion of hyaluronic acid has been proposed to drive propulsion of the non-sensory epithelium during canal morphogenesis, a process shown to be necessary for proper semicircular canal formation in Xenopus embryos [13]. The zebrafish ortholog of the human deafness gene *dfna5*, was subsequently shown to be necessary for *ugdh *expression and proper semicircular canal and jaw development [14]. Another zebrafish mutant, *gallery*, displays only a lateral semicircular canal protrusion and an immature anterior protrusion [15]. Although the *gallery *gene has not yet been identified, the defects in this mutant are believed to be caused by overexpression of Bmp4 (bone morphogenetic protein 4) in the presumptive cristae.

Several other genes required for proper semicircular canal formation in zebrafish have recently been identified. These include *sparc*, a gene encoding a matricellular glycoprotein (osteonectin) that functions in the genetic network regulating pharyngeal and inner ear development [16], *tmie *(transmembrane inner ear), a gene coding for a protein of unknown function that is important for normal hearing [17], and the Na, K-ATPase α subunit gene *α1a.2 *[18].

We have recently found that in zebrafish, the gene encoding neuronal calcium sensor-1a (*ncs-1a*) is required for semicircular canal formation [19]. NCS-1 is the mammalian ortholog of Drosophila frequenin, a protein critical for synaptic transmission within the Drosophila nervous system [20]. In mammalian cells, NCS-1 has been shown to promote exocytosis from dense core vesicles in both neurons and neuroendocrine cells [21], and plays a key role in G-protein coupled receptor desensitization via a direct interaction with the D2 dopamine receptor [22]. In zebrafish, morpholino knockdown of *ncs-1a *blocks normal development of the non-sensory portion of the semicircular canals [19]. However, the biochemical function of NCS-1 in regulating semicircular canal formation has not been elucidated.

To gain a better understanding of the role of NCS-1 in regulating inner ear development, we initiated a proteomic screen to identify NCS-1 binding partners (NBPs). Using yeast two-hybrid and bioinformatic approaches, we identified a cohort of human NBPs and their zebrafish orthologs. We examined expression of ten NBPs and identified seven that are expressed in the developing zebrafish inner ear. Based on their functional profiles, the hypothesis is presented that Ncs-1a/Pi4kβ/Arf1 form a signaling pathway that is required for semicircular canal formation. A second set of NBPs, consisting of *pink1*, *hint2*, and *slc25a25*, are destined for localization in mitochondria. Using antisense morpholinos, we show that *pink1 *is also required for proper development of the semicircular canals. Our findings reveal a cohort of novel intracellular NBPs. NCS-1/NBP interactions are likely to contribute to the molecular mechanisms that regulate the development of the vestibular apparatus.

## Results

### Identification of NCS-1 Binding Proteins (NBPs)

To identify NCS-1 binding proteins (NBPs), we used full-length human NCS-1 as bait in a Y2H screen of an adult human brain cDNA library. Candidate NCS-1 binding partners are listed in Table 1. Based on their functional properties we chose to pursue DAN (differential screening-selected gene aberrant in neuroblastoma), a secreted Bmp antagonist that has previously been shown to play a role in semicircular canal formation [23]; VAMP2, a synaptosomal protein involved in exocytosis [24-26]; and the mitochondrial proteins HINT2, SLC25A25 (SCAMC2), and PINK1, a mitochondrial kinase which has been found to be mutated in inherited forms of Parkinson's disease [27]. Additional interacting proteins identified in the Y2H screen were not examined further in this study. By conducting literature and protein database searches, we also identified several previously characterized NBPs (Table 1) whose potential role in ear development was analyzed. These include the ADP-ribosylation protein ARF1 [28], PI4Kβ [29], the inositol 1,4,5 triphosphate receptor IP3R [30], and TRPC (transient receptor potential channel) subtypes 1 [31] and 5 [32].

**Table 1:** Identification, cloning, and function of NBPs

**Human Gene**	**Gene Name**	**Function**	**Interaction**	**Zebrafish Ortholog**	**mRNA Accession Number (riboprobe NTs)**
Arf1	ADP-ribosylation factor 1	Trafficking/Exocytosis	GST-pulldown [28,42]	NM_201452	NM_201452 (99–643)

Pi4kβ (Pik4cb)	Phosphatidylinositol 4-kinase beta	Trafficking/Exocytosis	Immuno-precipitation [29]	BM095643	FJ032032 (1–2083)

Vamp2	Vesicle associated membrane protein 2/Synaptobrevin 2	Exocytosis	Y2H	BC059626	NM_200005 (1–894)

Hint2	Histidine triad protein	Mitochondrial AMP-lysine hydrolase	Y2H	CN504819	FJ032031 (1–514)

Slc25a25 (SCaMC2)	Ca^2+^-dependent mitochondrial carrier	Mitochondrial ATP-Mg/P_i _carrier	Y2H	NW_001513403	NM_213257 (65–1249)

Pink1	PTEN-induced kinase	Mitochondrial Kinase	Y2H	NW_001513662	FJ032033 (1–1725)

Trpc1	Classical transient receptor potential-1	Cation channel	GST-pulldown [31]	EE302043	XM_694363.3 (1097–2334)

Trpc5	Classical transient receptor potential-5	Cation channel	Immuno-precipitation and GST-pulldown [32]	AW343190	NM_001044827 (1–2395)

Ip3r (Itpr)	Inositol 1,4,5 trisphosphate receptor	Ca^2+ ^release channel	Immuno-precipitation [30]	CK017406	XM_691322 (6591–8187)

Dan (Nbl-1)	Differential screening-selected gene aberrant in neuroblastoma	Extracellular BMP antagonist	Y2H	BC066387	FJ032030 (1–702)

By using BLAST searches of the zebrafish EST database and genomic sequences available from GenBank and the Zebrafish Sequencing Group at the Sanger Institute  we identified zebrafish orthologs of each of the ten human NBPs. The sources and accession numbers for each of the zebrafish orthologs of the human NBPs are listed in Table 1. Zebrafish ESTs were obtained from ATCC (Manassas, VA). In cases where ESTs contained partial sequences, full-length ORFs (open reading frames) were generated by RT-PCR. Alignments of the predicted amino acid sequences of human NBPs and their zebrafish orthologs are presented in Additional File 1.

### Expression of NBPs in Zebrafish Inner Ear

**Figure 1:**
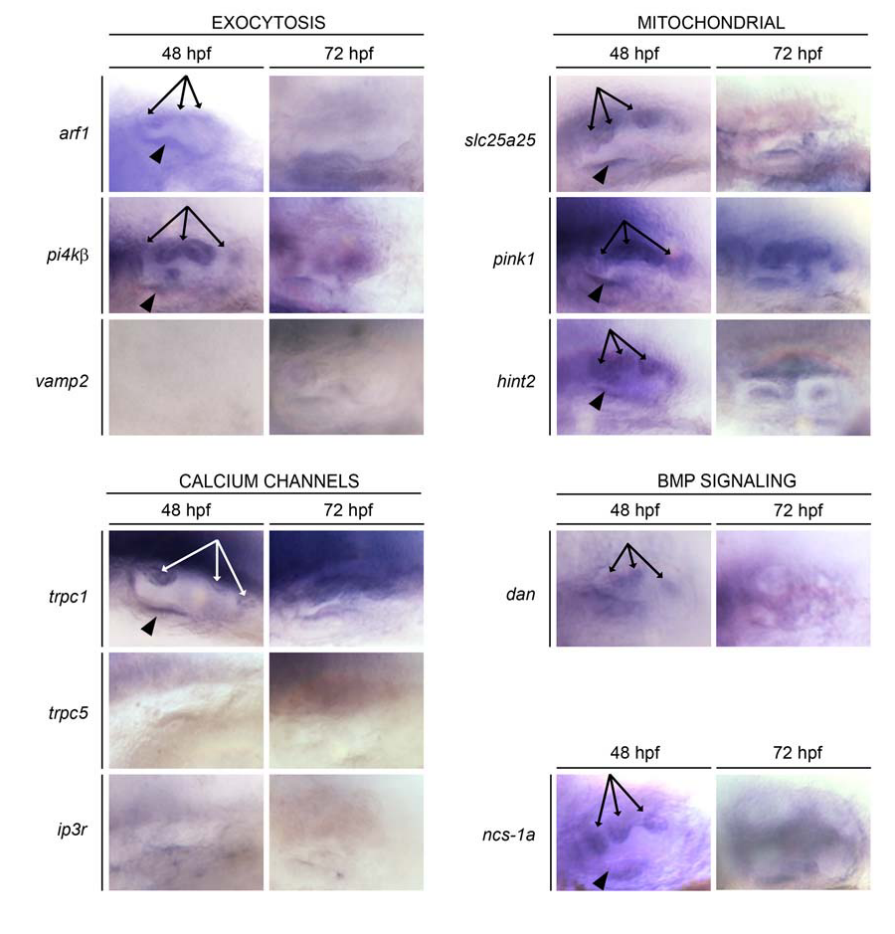
**Expression of NCS-1 binding partners (NBPs) during zebrafish inner ear development**. Whole mount in situ hybridization analysis was performed at 48 and 72 hpf. Genes are grouped according to presumed functional properties. All images are lateral views of the otic vesicle, anterior to the left. Arrows indicate mRNA expression in semicircular canal protrusions at 48 hpf. Arrowheads indicate mRNA expression in the anterior macula at 48 hpf.

We used whole-mount *in situ *hybridization to analyze mRNA expression for each of the 10 NBPs (listed in Table 1) during zebrafish otogenesis. The otic expression profile of each candidate NBP gene at 48 hpf and 72 hpf is shown in Fig. 1, while additional file 2 shows expression of these genes at 24, 48 and 72 hpf in the head region. Genes were grouped according to their known functional properties (i.e. exocytosis, calcium channels, Bmp signaling) or intracellular localization (i.e. mitochondria), and their otic expression profiles compared with that of the *ncs-1a *gene (Fig. 1: lower right panel). All of the genes investigated were expressed within the brain at each time point examined, while some were detected at high levels in additional structures including branchial arches (*arf*1, *pi4kβ*, *dan*, *ip3r*, *hint2*, *pink1*, and *slc25a25*), somites (*hint2*, *pink1*, and *slc25a25*), and heart (*ip3r*). Similarly, *ncs-1a *expression was detected throughout the brain and in the posterior somites and pharyngeal pouches [19].

At 48 hpf, *ncs-1a *transcripts can be visualized in the semicircular canal protrusions, and in the fully formed semicircular canal hubs at 72 hpf. We first analyzed expression of *arf1*, *pi4kβ*, and *vamp2*, NBP genes encoding proteins involved in exocytosis and intracellular trafficking. *arf1 *transcripts were expressed at 48 hpf in the epithelial protrusions of the semicircular canals but were not present in the semicircular canal hubs at 72 hpf, suggesting that *arf1 *contributes to early events in semicircular canal formation. Expression of *pi4kβ *mRNA, on the other hand, was very similar to the expression profile seen for *ncs-1a *mRNA. *pi4kβ *transcripts were detected at 48 hpf in the semicircular canal protrusions and in the fully formed semicircular canal hubs at 72 hpf. *vamp2 *mRNA was undetectable in the inner ear at either time point, indicating that *vamp2 *is unlikely to play a role in otogenesis. Three NBP genes, *pink1, hint2*, and *slc25a25*, each encoding a mitochondrial protein, showed very similar expression patterns. Transcripts of each gene were detected at 48 hpf in the semicircular canal protrusions and the anterior macula, and at 72 hpf in the semicircular canal hubs.

Amongst the genes encoding calcium channels, *trpc1 *transcripts were detectable at 48 hpf and 72 hpf in the semicircular canal protrusions and hubs, respectively. Transcripts of the other two calcium channel genes, *trpc5 *and *ip3r*, were undetectable in the inner ear at either 48 hpf or 72 hpf. Expression of *dan*, a gene encoding a secreted protein involved in Bmp signaling, was similar to that of the *pi4kβ *gene and was present in the semicircular canals at both 48 hpf and 72 hpf.

Taken together, our data indicate that seven NBP genes including *arf1*, *pi4kβ*, *trpc1*, *dan, pink1*, *hint2*, and *slc25a25*, are expressed in the semicircular canals during zebrafish embryogenesis and are likely to contribute to the formation of the vestibular apparatus.

### Validation of NCS-1/NBP Interactions

**Figure 2:**
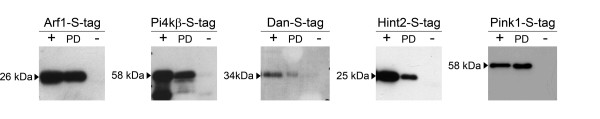
**GST-pulldowns**. GST pulldowns were performed to validate NCS-1/NBP interactions. A human NCS-1-GST fusion protein was used to pull down S-tagged zebrafish NBPs. In each panel, the left (+) lane contains a bacterial lysate expressing the designated S-tagged NBP, the middle lane (PD), contains NBPs pulled down from bacterial lysates after incubation with the NCS-1-GST fusion protein, and the right lane (-) shows proteins pulled down from lysates after incubation with GST alone. Blots were probed with an HRP-conjugated S-tag antibody to detect each S-tagged NBP. Molecular weights (kDa) of S-tagged NPB fragments are indicated to the left of each panel.

We used pulldown techniques to confirm the interaction between NCS-1 and several of the NBPs expressed in ear. Lysates from bacteria expressing S-tagged zebrafish proteins Arf1 (residues 1–180), Dan (residues 1–183), Pi4kβ (residues 1–423), Pink1 (residues 1–574), or Hint2 (residues 1–161) were tested for the ability to associate with an NCS-1-GST fusion protein containing full-length (residues 1–190) human NCS-1. In this context it is important to note that zebrafish *ncs1a *and human NCS-1 differ at only 8 of 190 positions [19]. Pulldowns were not performed for Slc25a25 or Trpc1. As shown in Fig. 2, a Western blot containing lysate from bacteria expressing S-tagged Arf1 produced an immunoreactive band of ~26 kDa when probed with an anti-S-protein antibody. This band corresponds to the expected size of the Arf1 fragment encoded by the cDNA construct. The same band was detected by pulldown after the bacterial lysate was incubated with the NCS-1-GST fusion protein, but not when the lysate was incubated with GST-containing beads alone. We next used the GST-pulldown assay to test whether NCS-1 also interacted with Dan, Pi4kβ, Hint2, and Pink1 (Fig. 2). Lysates from bacteria expressing S-tagged fragments of Dan (~34 kDa), Pi4kβ (~58 kDa), Hint2 (~25 kDa), and Pink1 (~58 kDa), each produced an immunoreactive band of the expected size (left lane in each panel). In each case, the same band was detected by pulldown after incubation with the NCS-1-GST fusion protein (middle lane of each panel). Virtually no immunoreactivity was detected when the lysates were incubated with beads containing GST alone (right lane in each panel). These results support the validity of the interaction between NCS-1 and Arf1, Dan, Pi4kβ, Pink1, and Hint2.

### Knockdowns of NBPs Involved in Exocytosis and Secretion

**Figure 3:**
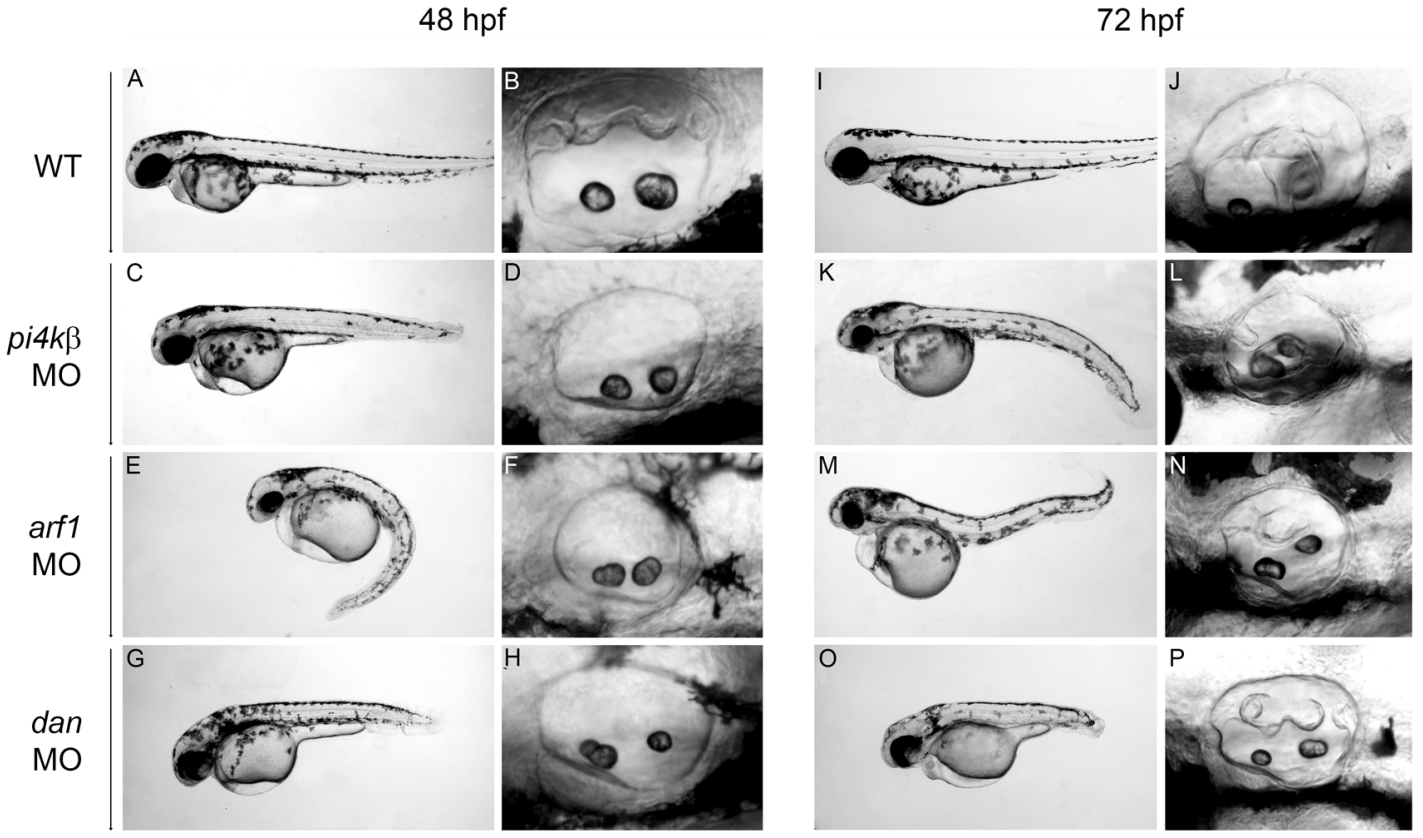
**Knockdown of *arf-1*, *pi4kβ*, and *dan *mRNA expression**. Antisense MOs targeted against *arf-1*, *pi4kβ*, or *dan *mRNA were injected into single cell embryos, and morphants analyzed at 48 (C-H) and 72 hpf (K-P). For comparison, control (buffer injected) embryos are shown at 48 (A-B) and 72 (I-J) hpf. Embryos were injected with either 4 ng *pi4kβ*-ATG MO (C-D, K-L), 2 ng *arf1*-UTR MO (E-F, M-N), or 1ng of *dan*-ATG MO (G-H, O-P). Images of otic vesicle (B, D, F, H, J, L, N, P) are all lateral views with anterior to the left. Lateral views of wild-type embryos are shown at 48 (A) and 72 (I) hpf. Lateral views of morphant embryos are shown at 48 (C, E, G) and 72 (K, M, O) hpf.

To gain an understanding of the function of NBPs during otogenesis, we used morpholino antisense oligonucleotides to knock down translation of selected NBP mRNAs in developing zebrafish. Our initial experiments involved knocking down translation of *arf1 *and *pi4kβ *mRNAs, encoding NBPs involved in exocytosis, and *dan *mRNA, encoding a secreted NBP known to play a role in semicircular canal formation [23]. We generated a set of non-overlapping morpholinos (MOs) targeted against either the initiating methionine (ATG-MO) or the 5'-UTR (UTR-MO) of *pi4kβ*, *arf1*, and *dan *mRNAs (Table 2), and microinjected the MOs into one cell-stage embryos. Representative examples of the NBP knockdowns are shown in Fig. 3. In wild type embryos, saccular and utricular otoliths are present at 48 hpf, and the semicircular canals protrusions are clearly visible (Fig. 3A). By 72 hpf, the epithelial pillars have fused to form the semicircular canal hubs exhibiting the characteristic cruciform pattern (Fig. 3J). Microinjection of MOs against each of the three NBP genes produced noticeable effects on inner ear development. MOs targeted against either *pi4kβ *(Fig 3C, K), *arf1 *(Fig. 3E, M), or *dan *(Fig. 3G, O), produced morphants with a shortened body axis, reduced eye size, and curved tails. These defects were most pronounced in *dan *morphants (Fig. 3G, O), even at low doses (1 ng) of the *dan *MO. At 48 hpf, semicircular canal protrusions were missing in > 90% of *pi4kβ *(Fig. 3D), *arf1 *(Fig. 3F), and *dan *(Fig. 3H) morphants (n = 50–60/group). Analysis of the developing ear through 48 hpf revealed that each of the NBP morphants exhibited normal otic placode induction and otolith formation compared to control embryos.

**Table 2:** Morpholino sequences

**Morpholino**	**Sequence**
*ncs1a*-MO	5'-TAGTTTGCTGTTGGATTTGCCCATC-3'
*arf1*-ATG MO	5'-TAAAGAGGTTTGCGAATATGTTTCC-3'
*arf1*-UTR MO	5'-CGCCTTGTTCACACCAAGTTCCAAG-3'
*pi4kβ*-ATG MO	5'-AAGCTCCAGCTCTGTATCACCCATG-3'
*pi4kβ*-UTR MO	5'-CACTTCAGGCCCCTCAAAATAAACC-3'
*dan*-ATG MO	5'-CGCGCACACACATCACCATACCTTC-3'
*dan*-UTR MO	5'-TCAGTGGATTAGCAGCTCGCGGTGT-3'
*pink1*-ATG MO	5'-GGCTGAGAACATGCTTTACTGACAT-3'

At 72 hpf, we observed that many of the morphants had begun to develop semicircular canals. However, the canals exhibited a range of aberrant morphologies including missing protrusions (Fig. 3L, N), failure of the pillars to fuse (Fig. 3P), as well as the presence of disorganized cell masses in the otic vesicle (Fig. 3L, N). For all genes tested, injection of a second, independent MO (either the ATG-MO or UTR-MO) phenocopied the ear defects generated by the primary MO (data not shown). In general, higher doses of the secondary MO were required to achieve effects comparable to those produced by the primary MO on semicircular canal formation. Together, these MO knockdown experiments provide compelling evidence that in addition to *ncs-1a*, expression of *arf1*, *pi4kβ*, and *dan *genes is required for semicircular canal formation in zebrafish.

### *ncs-1a*, *pi4kβ*, and *arf1 *Functionally Interact to Regulate Formation of Semicircular Canals

**Figure 4:**
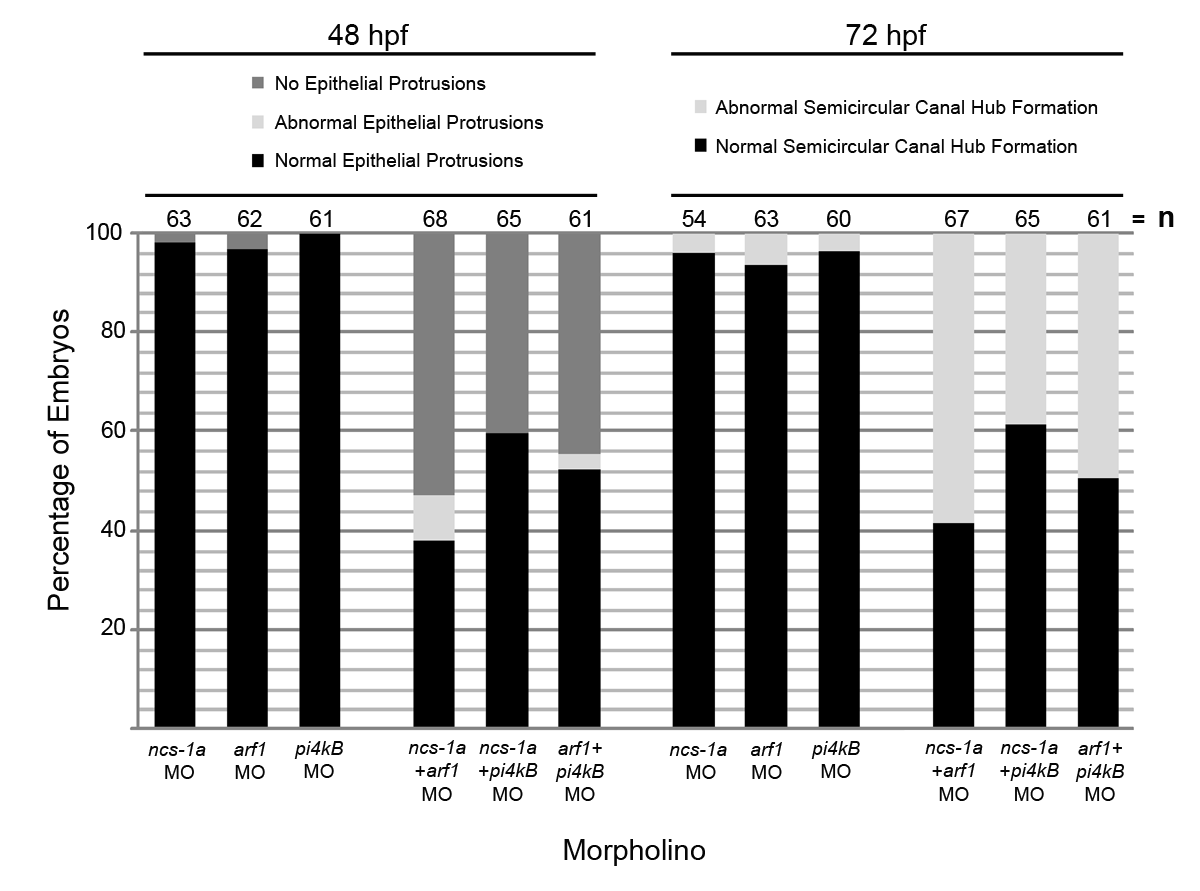
**Phenotypes of embryos co-injected with subeffective doses of NBP MOs**. Bar graph depicts percent of fish at 48 hpf or 72 hpf that displayed normal epithelial protrusions or semicircular canal hub formation, abnormal epithelial protrusions or semicircular canal hub formation, or absence of epithelial protrusions. Embryos were injected with 0.5 ng of *ncs-1a*-ATG MO alone, 0.5 ng of *arf1*-UTR MO alone, or 2 ng of *pi4kβ*-ATG MO alone. Alternatively, embryos were co-injected with 0.5 ng of *ncs-1a*-ATG MO plus 0.5 ng of *arf1*-UTR MO, 0.5 ng of *ncs-1a*-ATG MO plus 2 ng of *pi4kβ*-ATG MO, or 0.5 ng *arf1*-UTR plus 2 ng *pi4kβ*-ATG MO. The number of fish assayed for each treatment is displayed above the bars.

NCS-1, Arf1, and Pi4kβ have been shown to functionally interact in PC12 cells to regulate neuronal secretion [28]. We wished to determine whether these three genes functionally interact within the zebrafish inner ear to regulate semicircular canal development. To test this idea, we co-injected subeffective doses of *ncs-1a*-ATG (0.5 ng) plus *arf1*-UTR (0.5 ng) MOs, *ncs-1a-*ATG plus *pi4kβ*-ATG (2 ng) MOs, and *arf1*-UTR plus *pi4kβ*-ATG MOs, and examined the effect on semicircular canal formation. The results are shown in Fig. 4. At a concentration of 0.5 ng, neither the *ncs-1a*-ATG MO nor the *arf1*-UTR MO produced severe semicircular canal development defects, although a small percentage of fish (< 5%) displayed no epithelial protrusions at 48 hpf. 100% of embryos injected with 2 ng of the *pi4kβ*-ATG MO exhibited normal semicircular canal development at 48 hpf. However, co-injection of subeffective doses of *ncs-1a*-ATG plus *arf1*-UTR MOs caused an absence of epithelial protrusions in 53% of embryos at 48 hpf. Co-injection of *ncs-1a-*ATG plus *pi4kβ*-ATG MOs caused an absence of epithelial protrusions in 40% of embryos, while co-injection of subeffective doses of *arf1*-UTR plus *pi4kβ*-ATG MOs blocked outgrowth of epithelial protrusions in 44% of embryos. At 72 hpf, < 7% of embryos injected with subeffective doses of either *ncs-1a*-ATG, *arf1-UTR*, or *pi4kβ*-ATG MOs alone exhibited abnormal semicircular canal hub formation. Abnormal semicircular canal hub formation was observed in 58% of embryos co-injected with subeffective doses of *ncs-1a *plus *arf1 *MOs, in 38% of embryos co-injected with *ncs-1a*-ATG plus *pi4kβ*-ATG MOs, and in 49% of embryos co-injected with subeffective doses of *arf1*-UTR plus *pi4kβ*-ATG MOs. Co-injection of each pair of MOs thus appears to amplify the semicircular canal defects observed when subeffective doses of either *ncs-1a-*ATG, *arf1-*UTR, or *pi4kβ*-ATG MOs are injected alone. The functional interaction observed in this assay, in addition to the demonstrated physical interaction of their encoded proteins, suggests that *ncs1a*, *arf1*, and *pi4kβ *may act in a common pathway leading to formation of the semicircular canal system.

### *pink1 *is Required for Semicircular Canal Formation

PINK1 was initially implicated as an NBP in a Y2H screen (Table 1), while the interaction of zebrafish *pink1 *with NCS-1 was verified via pulldown with an NCS-1-GST fusion protein (Fig. 2). Whole-mount *in situ *hybridization analysis revealed expression of *pink1 *mRNA sequences in the epithelial pillars and hubs of the semicircular canals during zebrafish otogenesis (Fig. 1). To directly determine whether *pink1 *expression was necessary for ear development, we knocked down *pink1 *mRNA translation with a *pink1-*ATG MO and examined the effect on semicircular canal formation.

**Figure 5:**
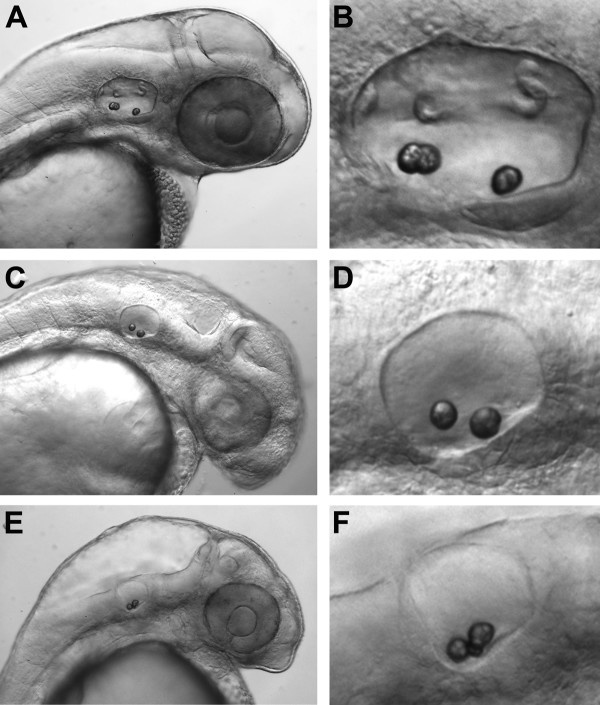
**Pink1 expression is required for semicircular canal development**. An antisense *pink1 *MO was used to knock down *pink1 *mRNA translation. Images are all lateral views of 48 hpf embryos with anterior to the right. (A) head of wild type embryo; (B) otic vesicle of wild type embryo. Head of *pink1 *morphant injected with 2 ng (C) or 4 ng (E) of *pink1*-ATG MO. Otic vesicle of *pink1 *morphant injected with either 2 ng (D) or 4 ng (F) of *pink1*-ATG MO.

A representative set of *pink1 *morphants is shown in Fig. 5. In control embryos at 48 hpf (Fig. 5A, B), otoliths are present and the epithelial protrusions of the semicircular canals are clearly visible. In 48 hpf MO-injected embryos, otoliths appear to have developed normally, although the overall size of the otocyst is somewhat smaller compared to control embryos. Microinjection of either 2 ng (Fig. 5C, D) or 4 ng (Fig. 5E, F) of a *pink1*-ATG MO produced morphants that were missing semicircular canal protrusions at 48 hpf in > 95% of embryos examined (n = 30). These results strongly support the idea that expression of the *pink1 *gene is required for semicircular canal formation in zebrafish.

## Discussion

Ncs-1 is a calcium sensing molecule that is required for semicircular canal formation in zebrafish [19]. To better understand of the role of NCS-1 in ear development, we employed a multistep functional proteomic strategy to identify molecular partners of NCS-1 that contribute to the development of the vestibular apparatus. First, by performing a Y2H screen in combination with literature and database searches, we identified 10 human NBPs. Second, zebrafish orthologs of each of these NBPs were identified using BLAST searches of the zebrafish EST and genomic databases. Third, by investigating the expression profiles of NBPs, we were able to deduce which candidate NBPs were likely to interact with *ncs-1a *in the ear and contribute to semicircular canal formation. We found that mRNA sequences encoding seven of the putative zebrafish NBPs were detected in the developing ear and exhibited an overlap in expression with that of the *ncs-1a *gene. On the other hand, three NBP genes (*vamp2*, *trpc5*, and *ip3r*) were not expressed in the ear, and thus were eliminated from further analysis. Fourth, GST pulldown experiments were carried out to confirm that candidate zebrafish NBPs also interacted with NCS-1. Finally, MO-mediated knockdowns confirmed that four selected NBPs were essential for semicircular canal formation in zebrafish.

**Figure 6:**
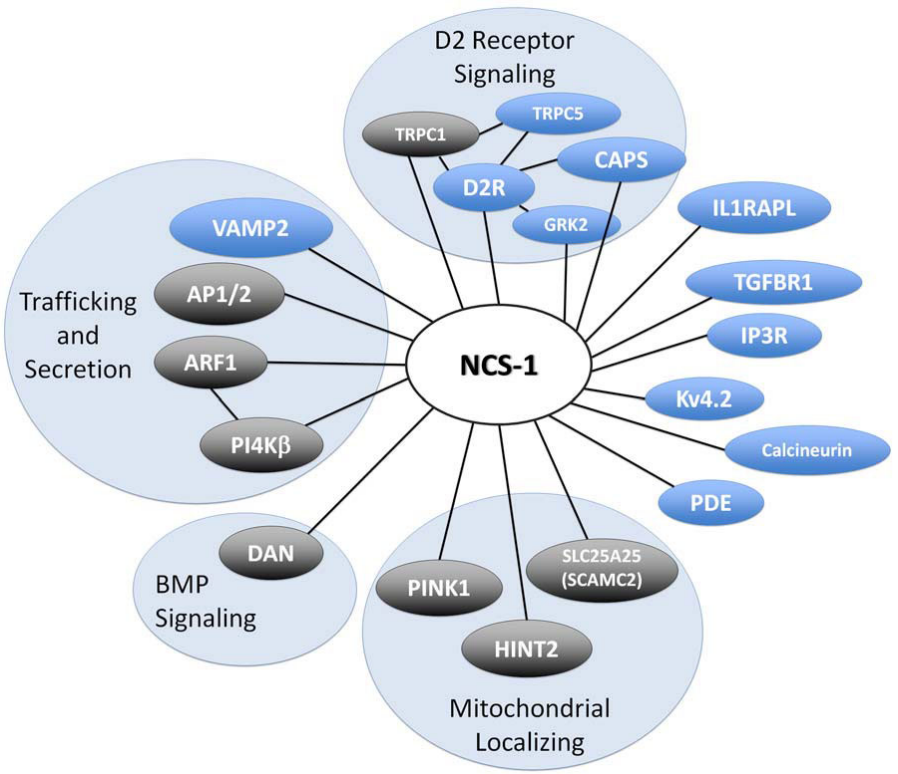
**NCS-1 interaction map**. Web of newly identified and previously known NCS-1 binding partners. Interaction map was adapted from Haynes et al. 2006 [33]. NBPs were grouped based on previously characterized functional properties or cellular localization. NBPs depicted in gray shaded ovals are those whose genes are expressed within the developing zebrafish inner ear.

The strategy outlined above has proven to be a fruitful method for identifying novel genes that contribute to inner ear development. Using this approach, we show that two previously identified (*arf1*, *pi4kβ*) and two novel (*dan*, *pink1*) NBP genes are required for semicircular canal formation (see interaction map, Fig. 6[33]). In the present study, we did not examine the role of *hint2*, *slc25a25*, or *trpc1 *using a morpholino knockdown approach. Further studies will be required to analyze the role of these and other NBPs in ear development. Mutagenesis screens in zebrafish combined with positional cloning approaches have traditionally been used to identify genes that contribute to ear development [8-10]. For example, the zebrafish *dog-eared *mutation was identified in a large-scale ENU screen by abnormal morphology of the inner ear [10]. The identity of this gene was then determined to be zebrafish *eya1 *via positional cloning [34]. While mutagenesis screens provide an unbiased strategy for gene discovery, this approach is generally extremely labor intensive and time consuming. Our strategy, on the other hand, circumvents some of the limitations inherent in an unbiased screen, by rapidly identifying interacting partners of genes already known to regulate inner ear development. This approach, in combination with the reverse genetic techniques available in zebrafish, should make it feasible to identify in a systematic fashion additional genes whose roles in ear development were previously unknown or overlooked.

The specificity of the phenotypes obtained by MO knockdown is supported by a number of considerations. First, morpholinos targeted against *pi4kβ*, *arf1*, and *dan *all caused similar phenotypes including vestibular malformation, shortened body axis, reduced head and eye size, and curved tails. Since each of these genes is expressed throughout the brain and jaw during embryonic development, defects in head formation were not unexpected. These defects are not likely due to off-target effects, since for each of the genes tested, two independent non-overlapping MOs produced identical phenotypes. Second, injection of buffer alone did not produce morphant phenotypes. Third, a MO targeted against otopetrin, a gene previously shown to be required for otolith formation [35], blocked development of otoliths but did not affect semicircular canal development. Fourth, injection of subeffective doses of *pi4kβ*, *arf1*, and *ncs-1a *MOs alone did not produce inner ear, jaw, or brain defects. Co-injection of subeffective doses of MOs against a pair of genes was synergistic and phenocopied the ear, jaw, and brain defects obtained using an effective dose of each of MO alone. Finally, brain and ear development appear normal in *pi4kβ*, *arf1*, and *dan *morphants until 48 hpf, indicating that disruption of early patterning events is not likely to have caused vestibular phenotypes or developmental delay. Together, these results support the idea that the phenotypes produced by NBP knockdowns are specific, and do not result from non-specific cytotoxic or off-target gene effects.

**Figure 7:**
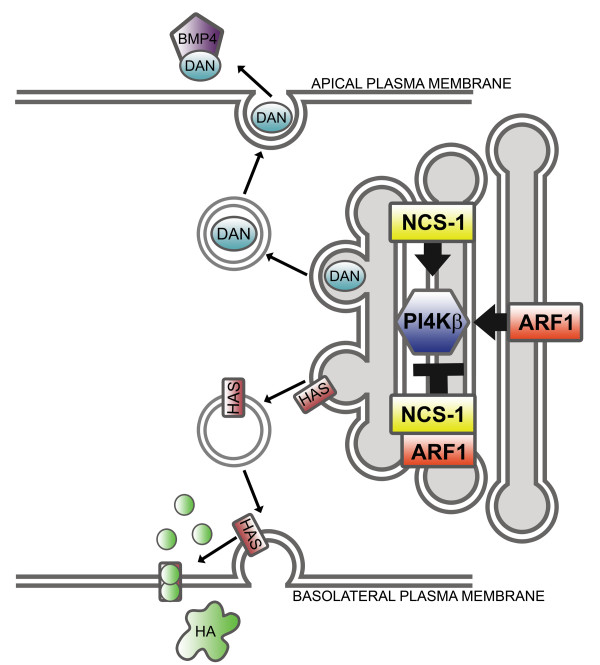
**Proposed model for functional interaction between Ncs-1a, Pi4kβ, and Arf1 in trafficking and secretion**. Dan, Bmp4, and HA are secreted molecules that contribute to proper semicircular canal development and are predicted to require the action of an Ncs-1/Pi4kb/Arf1 signaling pathway for their secretion. Bold arrows indicate activation, whereas the bold T indicates inhibition, of this signaling pathway. Thin arrows indicate directional flow of vesicles or proteins.

Our double MO knockdown studies, together with the pulldown experiments, indicate that NCS-1, Arf1 and Pi4kβ form a small interaction network (Fig. 6 adapted from [33]) that is likely to have functional significance for development of the zebrafish semicircular canal system. We now propose a model in which these three proteins regulate the targeted secretion of molecules, such as Dan, that are required for formation of the semicircular canals (Fig. 7). This model is based on the following lines of evidence. Pi4kβ is the enzyme responsible for the synthesis of phosphatidylinositol 4-phosphate, a lipid species that regulates trafficking of cargo from the Golgi apparatus to the plasma membrane in yeast and mammalian cells [36-38]. Pi4kβ has also been shown to regulate secretion in both PC12 cells and pancreatic β cells [39,40]. NCS-1 [41] and Arf1 [41] are known activators of Pi4kβ, however a direct interaction between NCS-1 and Arf1 has been shown to antagonize the enzymatic activity of Pi4kβ [28,42]. Bidirectional control of Pi4kβ activation/inhibition may thus serve to regulate the trafficking of molecules involved in inner ear development from the trans-golgi network to the plasma membrane during otogenesis.

Dan, Bmp4, and Hyaluronic acid represent secreted molecules that play critical roles in semicircular canal formation [13-15,23,43] and may require an *ncs-1a*/*arf1*/*pi4kβ *pathway for secretion. BMPs are expressed in the developing vertebrate inner ear and participate in axial patterning of the otic vesicle and differentiation of the sensory epithelium [23,44-48]. Bmp4 is secreted from the apical membrane of cells located in the cristae of the otic vesicle and its regulated expression is essential for proper development of the non-sensory portion of the semicircular canals [15,43]. Dan is a Bmp antagonist that plays a key role in the patterning of the chick inner ear, and is also secreted from the apical membrane [23]. In chick, loss of Dan leads to structural deformities in the semicircular canals and the endolymphatic duct and sac. The MO knockdown experiments we performed show that Dan also contributes to semicircular canal formation in zebrafish. We have also demonstrated a direct interaction between Dan and NCS-1. Although the significance of this interaction is currently unknown, it is possible that the Dan/NCS-1 interaction may be required to deliver Dan to the Golgi apparatus. This idea is supported by previous studies which showed that the yeast ortholog of NCS-1 (Frq1p) can recruit Pik1p (yeast ortholog of PI4kβ) from the endoplasmic reticulum to the trans-Golgi network [49].

Hyaluronic acid (HA) is an extracellular matrix molecule that is secreted from the basolateral membrane of otic epithelium during semicircular canal formation and is thought to drive the outgrowth of the developing protrusions [13]. Hyaluronan synthases (HAS) are the enzymes responsible for HA synthesis and secretion at the plasma membrane [50]. It is possible that trafficking of HAS from the Golgi apparatus to the plasma membrane may also be regulated by Pi4kβ activity. Since HA secretion from epithelial pillars in zebrafish inner ear can be monitored histochemically [14], it should be possible to test this hypothesis by determining whether knockdown of Ncs-1a, Arf1, or Pi4kβ inhibits HA secretion.

Our yeast two-hybrid screen revealed interaction of NCS-1 with three proteins, HINT2, SLC25a25, and PINK1, that are destined to localize to mitochondria. The interaction of NCS-1 with Pink1 *(*phosphatase/tensin homolog on chromosome 10 (PTEN)- induced putative kinase 1) is of particular interest. Mutations in the human PINK1 gene are the second most common cause of autosomal recessive Parkinson's disease [51], and are also implicated in sporadic cases of the disease [52]. MO knockdown studies we performed indicate that *pink1 *morphants exhibit severe brain abnormalities as well as defects in development of the semicircular canal system. Interestingly, a recent study in zebrafish also showed that *pink1 *morphants display severe defects in brain morphology, as well an impaired escape response [53]. In this context, it is important to consider that no direct link has been established between mutations in Pink1 and defects in inner ear development in mammalian systems. It is therefore possible that Pink1 has evolved to perform a critical role in brain and ear development in zebrafish, while mammals may have developed compensatory mechanisms that regulate development of these organs.

The interaction of NCS-1 with zebrafish Pink1 raises another interesting issue. NCS-1 does not possess a defined mitochondrial targeting sequence, and its expression in mitochondria has not been documented. In mammalian cells, NCS-1 is constitutively associated with the plasma membrane and Golgi complex [54]. It will be important to determine whether NCS-1 serves to regulate Pink1 function, either within mitochondria or other cellular compartments, and whether the NCS-1/Pink1 interaction contributes to the etiology of Parkinson's disease.

## Conclusion

In this study, we show that several new and previously identified NBPs appear to play an important role in the development of the zebrafish inner ear. We suggest a model in which Ncs-1a/Arf1/Pi4kβ interactions may form a signaling pathway that mediates trafficking and export of secreted molecules that are required for semicircular canal formation. We also made an unexpected observation that NCS-1 interacts with several mitochondrial proteins, including Pink1, a gene that contributes to familial Parkinson's disease. These findings suggest a previously unrecognized role for NCS-1 in mitochondrial function via its association with mitochondrial proteins, and reveal a new and potentially important signaling pathway involved in development of the vestibular apparatus.

## Methods

### Yeast two-hybrid Screen

A yeast two-hybrid (Y2H) screen was performed as previously described [55] using human NCS-1 as bait to screen an adult human brain cDNA library. Full length human NCS-1 was subcloned into the yeast GAL4 DNA-binding domain expression vector pAS2-1 (Clontech, Palo Alto, CA), while the human brain cDNA library was subcloned into the GAL4 activation domain vector pACT2 (Clontech). Bait and prey plasmids were simultaneously co-transformed into yeast strain MaV103 [55]. Positive clones were identified by growth on Leu^-^/Trp^-^/His^-^/Ura^- ^selection plates. Protein interaction was assayed for by β-galactosidase activity via the nitrocellulose filter lift method [55]. cDNAs were extracted from yeast colonies, sequenced, and subjected to a BLAST search against the human genome to determine the identity of the NCS-1 interacting cDNA clones.

### Identification and Cloning of Zebrafish NBP Orthologs

Zebrafish orthologs of human NBPs were identified by using BLAST searches of the zebrafish EST database and genomic sequences available from GenBank  and the Zebrafish Sequencing Group at the Sanger Institute . The sources and accession numbers for each of the zebrafish orthologs of the human NBPs are listed in Table 1. Zebrafish ESTs were obtained from ATCC (Manassas, VA). For RT-PCR (reverse transcriptase-polymerase chain reaction), 48 hpf (hours post fertilization) zebrafish embryos were collected and homogenized in TRIzol Reagent (Invitrogen; Carlsbad, CA). Total RNA was extracted as previously described [56], and 0.5 μg of RNA was used as template to generate single stranded cDNA using the SuperScript First Strand Synthesis kit (Invitrogen), and RT-PCR performed using REDTaq DNA polymerase (Sigma; St. Louis, MO) and a RoboCycler Gradient Temperature Cycler (Stratagene; La Jolla, CA). Primers used for RT-PCR are listed in Table 3.

**Table 3:** Primers for cloning NBPs

**Zebrafish Gene**	**RT-PCR Forward Primer**	**RT-PCR Reverse Primer**
*arf1*	5'-GATGGGAAACATATTCGCAAACCT-3'	5'-TCATTTCTGGTTTTTCAGCTGATTGGACA-ACC-3'

*pi4kβ*	5'-GTGGCCATGGGTGATACAGAGCTGGAG--CTTTCTC-3'	5'-TCACATGATACCGTTGGTCAGATACTGG-AAGCCGT-3'

*hint2*	5'-ACGGTCACGAACGTAGTTTGTCC-3'	5'-CTATCCTGGAGGCCATTTCATTTGCC-3'

*pink1*	5'-CAGATGACAGTGGAGGAGCTTCTGC-3'	5'-CTATGGCTGAGAGTTAGACATCAGCAGGT-ATC-3'

*trpc1*	5'-CTCTCTCCGTTATAATGGCTGCTC-3'	5'-GCTCAGCTGGTTCCGTGACTCTTC-3'

*dan*	5'-TCAGCATCTGAGATCCGTCAGCGGGAC-GTC-3'	5'-GTTTCACCCAGCCTCGGGCGCGTGCAT-GTC-3'

### Whole-mount in situ Hybridization

48 and 72 hpf zebrafish embryos were preserved in 4% paraformaldehyde in PBS. Whole-mount *in situ *hybridization analysis was performed as previously described [57]. Riboprobes were synthesized for the following genes (Table 1): *arf1, dan*, *hint2*, *ip3r*, *pink1*, *pi4kβ, slc25a25, trpc1*, *trpc5*, and *vamp2*.

### Morpholino Knockdowns

Antisense morpholino oligonucleotides (MOs) were obtained from Gene Tools LLC (Philomath, OR). Two non-overlapping MOs were targeted against either the initiating methionine or the 5' untranslated region of *pi4kβ*, *arf1*, *ncs*-*1a*, *dan*, and *pink1 *mRNAs. The sequence for each morpholino is listed in Table 2. MOs were resuspended in 1× Danieau buffer (58 mM NaCl, 0.7 mM KCl, 0.4 mM MgSO_4_, 0.6 mM Ca(NO_3_)_2_, 5 mM HEPES, pH 7.6), and microinjected directly into the yolk sac of single cell embryos.

### Glutathione S-transferase Pulldown

An NCS-1-GST (glutathione S-transferase) fusion protein was constructed by fusing amino acids 1–190 of human NCS-1 to GST in the expression vector pGEX-4T-1 (Amersham Biosciences, Piscataway, NJ). The NCS-1-GST-fusion protein was induced in *Escherichia coli *strain BL21 (DE3) using the ZYP-5052 auto-induction media described previously [58,59], then purified on glutathione sepharose beads (Amersham) according to manufacturer's instructions. cDNAs encoding zebrafish *arf1 *(amino acids 1–180), *pi4kβ *(residues 1–423), *dan *(residues 1 – 183), *pink1 *(residues 1–574), and *hint2 *(residues 1–161) were subcloned into the pET30C expression vector containing an S-tag. Constructs were induced using auto-induction media as described above. NCS-1-GST fusion protein was used to pull down NBPs from bacterial lysates as previously described [60]. Eluted proteins were separated by SDS-PAGE and transferred to a PVDF (polyvinylidene fluoride) filter. The filter was probed with a horseradish peroxidase conjugated S-tag antibody (1:5000 dilution, Santa Cruz Biotechnology). Immunoreactivity was detected by enhanced chemiluminescence with an ECL Plus kit (Amersham).

## Abbreviations

NBP: NCS-1 binding protein; GST: glutathione S-transferase; HPF: hours post fertilization; HA: hyaluronic acid; Y2H: yeast 2 hybrid; EST: expressed sequence tag; RT-PCR: reverse transcriptase polymerase chain reaction; ORF: open reading frame; cDNA: copy DNA; MO: morpholino; ENU: N-ethyl-N-nitrosourea; PVDF: polyvinylidene fluoride; SDS-PAGE: sodium dodecyl sulfate polyacrylamide gel electrophoresis.

## Authors' contributions

JAP identified and generated sequence alignments of the zebrafish orthologs of human NBP's, performed all molecular cloning procedures, in situ hybridizations, GST-pulldowns, and morpholino knockdowns, and drafted the manuscript. NK performed the yeast two-hybrid screen and assisted in the preparation of the manuscript. CF, MW, KH, and MC assisted with GST-pulldowns. VAC assisted with bioinformatics, identification of zebrafish NBP orthologs, and preparation of the manuscript. RL conceived of the study, and participated in its design and coordination and helped to draft the manuscript. All authors read and approved the final manuscript.

## Supplementary Material

Additional File 1 **Comparison of human and zebrafish NBPs. Amino acid alignments of the human (h) and zebrafish (zf) NBP orthologs listed in Table **1. Identical amino acids are highlighted in black, and conserved amino acids are highlighted in gray. Amino acids are numbered to the left. File is a pdf and can be viewed with adobe acrobat reader. Genbank accession numbers for sequences used in the alignments are as follows: h_Arf1 (CAI23120), zf_Arf1 (NP_958860), h_Dan (BAA92265), zf_Dan (ACH92116), h_Hint2 (CAI10991), zf_Hint2 (ACH92117), h_Ip3r (NP_002215), zf_Ip3r (XP_696414), h_Pi4kβ (AAH00029), zf_Pi4kβ (ACH92118), h_Pink1 (AAQ89316), zf_Pink1 (ACH92119), h_Slc25a25 (CAI13838), zf_Slc25a25 (NP_998422), h_Trpc1 (CAA61447), zf_Trpc1 (XP_699455), h_Trpc5 (NP_036603), zf_Trpc5 (NP_001038292), h_Vamp2 (NP_055047), zf_Vamp2 (NP_956299). Click here for file

Additional File 2 **Expression of zebrafish NBPs in head region. **Whole mount in situ hybridization analysis was performed at 24, 48, and 72 hpf. Expression profiles in head region are shown for all of the NBPs represented in Figure 1. Genes are grouped according to presumed functional properties. All images are lateral views of the head, anterior to the left. Click here for file
