# Supplementary material for: Proteomic and functional analysis of NCS-1 binding proteins reveals novel signaling pathways required for inner ear development in zebrafish
Source: BMC Neurosci. 2009 Mar 25;10:27. doi: 10.1186/1471-2202-10-27 (PMC2679751; doi:10.1186/1471-2202-10-27)
Supplement: Additional File 1 — Comparison of human and zebrafish NBPs. Amino acid alignments of the human (h) and zebrafish (zf) NBP orthologs listed in Table 1. Identical amino acids are highlighted in black, and conserved amino acids are highlighted in gray. Amino acids are numbered to the left. File is a pdf and can be viewed with adobe acrobat reader. Genbank accession numbers for sequences used in the alignments are as follows: h_Arf1 (CAI23120), zf_Arf1 (NP_958860), h_Dan (BAA92265), zf_Dan (ACH92116), h_Hint2 (CAI10991), zf_Hint2 (ACH92117), h_Ip3r (NP_002215), zf_Ip3r (XP_696414), h_Pi4kβ (AAH00029), zf_Pi4kβ (ACH92118), h_Pink1 (AAQ89316), zf_Pink1 (ACH92119), h_Slc25a25 (CAI13838), zf_Slc25a25 (NP_998422), h_Trpc1 (CAA61447), zf_Trpc1 (XP_699455), h_Trpc5 (NP_036603), zf_Trpc5 (NP_001038292), h_Vamp2 (NP_055047), zf_Vamp2 (NP_956299). [file 1471-2202-10-27-S1.pdf]

```
h_Arf1      1  MGNIFANLFKGLFGKKEMRILMVGLDAAGKTTILYKLKLGEIVTTIPTIGFNVETVEYKN
zf_Arf1     1  MGNIFANLFK-VFGKKEMRILMVGLDAAGKTTILYKLKLGEIVTTIPTIGFNVETVEYKN


h_Arf1     61  ISFTVWDVGGQDKIRPLWRHYFQNTQGLIFVVDSNDRERVNEAREELMRMLAEDELRDAV
zf_Arf1    60  ISFTVWDVGGQDKIRPLWRHYFQNTQGLIFVVDSNDRERVNEAREELMRMLAEDELREAV


h_Arf1    121  LLVFANKQDLPNAMNAAEITDKLGLHSLRHRNWYIQATCATSGDGLYEGLDWLSNQLRNQ
zf_Arf1   120  LLVFANKQDLPNAMNAAEITDKLGLHSLRHRNWYIQATCATSGDGLYEGLDWLSNQLKNQ


h_Arf1    181  K
zf_Arf1   180  K
```

```
h_Dan      1 ---MLRVLVGAVLPAMLLAAPPP-INKLALFPDKSAWCEAKNITQIVGHSGCEAKSIQNR
zf_Dan     1 MVMCVRAVLVCVLLELSRAAPHAHINRLALFPDKSAWCEAKNITQIVGHTGCTPRSIQNR

h_Dan     57 ACLGQCFSYSVPNTFPQSTESLVHCDSCMPAQSMWEIVTLECPGHEEVPRVDKLVEKILH
zf_Dan    61 ACLGQCFSYSVPNTFPQSTESLVHCDSCMPAQTQWEVVTLDCSGSDEAPRVDKLVERILH

h_Dan    117 CSCQACGKEPSHEGLSVYVQGEDGPGSQPGTHPHPHPHPHPGGQTPEPEDPPGAPHTEEE
zf_Dan   121 CSCQSCSKESSQEGALLQLYPHEGAQDLPSLPDATHTHPQHAHMQADQRD--ADAHLDMH

h_Dan    177 GAED-
zf_Dan   179 APEAG
```

```
h_Hint2      1 -MAAAVVLAAGLRAARRAVAATGVRGGQVRGAAGVTDGNEVAKAQQATP--GGAAPTIFS
zf_Hint2     1 MTACGVLLTRQLLPFGRGLWRVLS----VKSCYSTIN-DEVRLAQEASKKYGKLEPTIFT

h_Hint2     58 RILDKSLPADILYEDQQCLVFRDVAPQAPVHFLVIPKKPIPRISQAEEEDQQLLGHLLLV
zf_Hint2    56 KIIDKTVPAVIIYEDDKCLAFRDVNPQAPVHYLVIPRIPIPRISEAHDEDSLILGHLLVV

h_Hint2    118 AKQTAKAEGLGDGYRLVINDGKLGAQSVYHLHIHVLGGRQLQWPPG
zf_Hint2   116 AKNIAKKEGLAEGYRVVINDGKNGAQSVYHLHIHVLGGRQMKWPPG
```

```
h_Ip3r     1 -MSEMSSFLHIGDIVSLYAEGSVNGFISTLGLVDDRCVVEPAAGDLDNPPKKFRDCLFKV
zf_Ip3r    1 MSDSASSFLHIGDIVSLYAEGTVNGFISTLGLVDDRCVVEPASGDLENPPKKFRDCLFKV

h_Ip3r    60 CPMNRYSAQKQYWKAKQTKQDKEKIADVVLLQKLQHAAQMEQKQNDTENKKVHGDVVKYG
zf_Ip3r   61 YPMNRYSAQKQFWKAKQAKHEKDKIGDMVLLQKLQHAANLEQKQNDAENKKVHGDVVKYG

h_Ip3r   120 SVIQLLHMKSNKYLTVNKRLPALLEKNAMRVTLDATGNEGSWLFIQPFWKLRSNGDNVVV
zf_Ip3r  121 SVIQLLHMKSNKYLTVNKRLPALLEKNAMRVTLDGTGNEGSWLFIQPFWKLRANGDNVVV

h_Ip3r   180 GDKVILNPVNAGQPLHASNYELSDNAGCKEVNSVNCNTSWKINLFMQFRDHLEEVLKGGD
zf_Ip3r  181 GDKVILNPVNAGQPLHASSYELPDHSGCKEINSVNSNTSWKISLFMMFSDHKDEVLKGGD

h_Ip3r   240 VVRLFHAEQEKFLTCDEYKGKLQVFLRTTLRQSATSATSSNALWEVEVVHHDPCRGGAGH
zf_Ip3r  241 VVRLFHAEQEKFLTCDEYKSKLHVFLRTTLRQSATSATSSNALWEVEVVHHDPCRGGAGH

h_Ip3r   300 WNGLYRFKHLATGNYLAAEENPSYKGDASDPKAAGMGAQGRTGRRNAGEKIKYCLVAVPH
zf_Ip3r  301 WNSLYRFKHLATGNYLAAEENPGYKENSNDIEVGYIVDSSRIKR-SLGERIKYKLVAVPH

h_Ip3r   360 GNDIASLFELDPTTLQKTDSFVPRNSYVRLRHLCTNTWIQSTNVPIDIEEERPIRLMLGT
zf_Ip3r  360 GNDIASLFELDPTTLQKTDSFVPRNSYVRFRHLCTNTWIQSTNIPIDIDEERPIRLMLGT

h_Ip3r   420 CPTKEDKEAFAIVSVPVSEIRDLDFANDASSMLASAVEKLNEGFISQNDRRFVIQLLEDL
zf_Ip3r  420 CPTKEDKEAFAIVSVPVMEIRDLDFANDASLMLSTVVEKFKYGFLSPNDRRYAIKLLEDV

h_Ip3r   480 VFFVSDVPNNGQNVLDIMVTKPNRERQKLMREQNILKQVFGILKAPFREKGGEGPLVRLE
zf_Ip3r  480 VFFVVDQLNNGQPALEVSMNKPNRERQKLMREQNILKQIFGIIKAPFKERGDDPPLLTLE

h_Ip3r   540 ELSDQKNAPYQHMFRLCYRVLRHSQEDYRKNQEHIAKQFGMMQSQIGYDILAEDTITALL
zf_Ip3r  540 ELSDQKYLLYQYMLRLCYRVLRHSQEDYRKNQEYIAKQFGMMQSQIGYDILAEDTITALL

h_Ip3r   600 HNNRKLLEKHITKTEVETFVSLVRKNREPRFLDYLSDLCVSNHIAIPVTQELICKCVLDP
zf_Ip3r  600 HNNRKLLEKHITKTEVETFVSLVRKNREPRFLDYLSDLCVSNNVAIPVTQELICKCVLNA

h_Ip3r   660 KNSDILIRTELRPVKEMAQSHEYLSIEYSEEEVWLTWTDKNNEHHEKSVRQLAQEARAGN
zf_Ip3r  660 KNQDILMKTERRAAKESQHTSEYLEEFGDEDEVWLIWSEKENENHEKSIRQLAQEARQGN

h_Ip3r   720 AHDENVLSYYRYQLKLFARMCLDRQYLAIDEISQQLGVDLIFLCMADEMLPFDLRASFCH
zf_Ip3r  720 VHDENVLTYYRYQLKLFARMCFDRQYLAIDVISKQLDIELISLCMMDETLPFDLRASFCR

h_Ip3r   780 LMLHVHVDRDPQELVTPVKFARLWTEIPTAITIKDYDSNLNASRDDKKNKFANTMEFVED
zf_Ip3r  780 LMLHAHVDRDPQELVTPIKFARLWTEIPTSISIKDYDSHLDYSRDNKKNKFANTMAFMEE
```

```
h_Ip3r    840 YLNNVVSEAVPFANEEKNKLTFEVVSLAHNLIYFGFYSFSELLRLTRTLLGIIDCVQGPP
zf_Ip3r   840 YLNNVLIDDLPFANEEKNKLTYEVVSLARHLIYFGFYSFFELLRLTRTLLGIIDCTPSNA

h_Ip3r    900 AMLQAYEDPGGKNVRRSIQGVGHMMSTMVLSRKQSVFSAPSLSAGASAAEPLDRSKFEEN
zf_Ip3r   900 SINPLFNDDGSKNVRRSIHGMGQMMSTMVLNRKPSLFSAP----GRTGDSQVGSKDSIDT

h_Ip3r    960 EDIVVMETKLKILEILQFILNVRLDYRISYLLSVFKKEFVEVFPMQDSGADGTAPAFDST
zf_Ip3r   956 QDITVMDTKLKILEILQFILSVRLDYRLSFLLSVFKKEFVDVYPMEDADAT-HHTEHEGN

h_Ip3r   1020 TANMNLDRIGEQAEAMFGVGKTSSMLEVDDEGGRMFLRVLIHLTMHDYAPLVSGALQLLF
zf_Ip3r  1015 RSSINLQHIGEQAEAMFGIGKGNSILEVDDEGGRMFLRVLIHLIMHDYPPLVSGALQLLF

h_Ip3r   1080 KHFSQRQEAMHTFKQVQLLISAQDVENYKVIKSELDRLRTMVEKSELWVDKKGSGKGEEV
zf_Ip3r  1075 KHFSQRQEVLHTFKQVQLLISTQDVDNYKHIKRDLDLLRTMVEKSELWVIKKSSSGGDGK

h_Ip3r   1140 EAGAAKDKKE----RPTDEEGFLHPPGEKSSENYQIVKGILERLNKMCGVGEQMRKKQQR
zf_Ip3r  1135 KD--KKDKKEPEAVSPEEEADSKKQTTEKSNESYQNVKEILERLNKMCSSG--VFKKQQR

h_Ip3r   1196 LLKNMDAHKVMLDLLQIPYDKGDAKMMEILRYTHQFLQKFCAGNPGNQALLHKHLHLFLT
zf_Ip3r  1191 LLKNMGAHKVMLDLLQISYDRNDTKMLEIIKYTHLFLQKFCTGNLENQALLHKHLNLFLT

h_Ip3r   1256 PGLLEAETMQHIFLNNYQLCSEISEPVLQHFVHLLATHGRHVQYLDFLHTVIKAEGKYVK
zf_Ip3r  1251 PRLLEAETMQQIFSNNFQLCSEISESVLHHFIHCLATQGRHIQYLNFLHTIIKAEGKYVK

h_Ip3r   1316 KCQDMIMTELTNAGDDVVVFYNDKASLAHLLDMMKAARDGVEDHSPLMYHISLVDLLAAC
zf_Ip3r  1311 KCQDMIMTELTSAGDDVVVLYTDDTSFNTMVELMTQSREGVKDDSPLRYHISLVELLAAC

h_Ip3r   1376 AEGKNVYTEIKCTSLLPLEDVVSVVTHEDCITEVKMAYVNFVNHCYVDTEVEMKEIYTSN
zf_Ip3r  1371 AEGKNVYTEIKCTSLLPLEEMVKVITHEDCITEVKIAYVNFVNHCYVDTEVEMKEIYTSN

h_Ip3r   1436 HIWTLFENFTLDMARVCSKREKRVADPTLEKYVLSVVLDTINAFFSSPFSENSTSLQTHQ
zf_Ip3r  1431 HIWKLFENFTVDMARVCSNREKRMSDPVLEKYVIQVVLDTVTAFFSSPFSENSTSTEAHH

h_Ip3r   1496 TIVVQLLQSTTRLLECPWLQQQHKGSVEACIRTLAMVAKGRAILLPMDLDAHISSMLSSG
zf_Ip3r  1491 TTVKQLLQSTMRLLDCPWLQPQQKVQVESCIRTLAVTTKSRSIPLPVELEAHVNMMLS--

h_Ip3r   1556 ASCAAAAQRNASSYKATTRAFPRVTPTANQWDYKNIIEKLQDIITALEERLKPLVQAELS
zf_Ip3r  1549 HSNSLTLSRSSHSNKSMSRLTRPAAPTN-PWDYKNIIEKLQDIINTLEERVMPLVNAELS

h_Ip3r   1616 VLVDVLHWPELLFLEGSEAYQRCESGGFLSKLIQHTKDLMESEEKLCIKVLRTLQQMLLK
zf_Ip3r  1608 VLVDVLHQPELLFLEGTDARSRCESGGFISKLIQHTKALMNSDEKLCIKVLRTLQEMLIR
```

```
h_Ip3r  1676 KTKYGDRGNQLRKMLLQNYLQNRKSTSRGDLPDPIGTGLDPDWSAIAATQCRLDKEGATK
zf_Ip3r 1668 ELDFDEKGFALRKVLLQNYLYNNKKNIKAELVEHAGEG-ERDWLTVAALQCRLDKEGGTK

h_Ip3r  1736 LVCDLITSTKNEKIFQESIGLAIHLLDGGNTEIQKSFHNLMMSDKKSERFFKVLHDRMKR
zf_Ip3r 1727 LFTDLITSTKNEKIFQESIQLAICLLEGGNTEIQNSFYKLMMGDNKSEKFFKVLNDRMKN

h_Ip3r  1796 AQQETKSTVAVNMNDLGSQPHEDREPVDPTTKGRVASFSIPGSSSRYSLGPSLRRGHEVS
zf_Ip3r 1787 AQLDIKSTVSVNVGEMSNKAKDDK---DLETGNRNNNFPSGGVSSFGQPEP------QPE

h_Ip3r  1856 ERVQSSEMGTSVLIMQPILRFLQLLCENHNRDLQNFLRCQNNKTNYNLVCETLQFLDIMC
zf_Ip3r 1838 QQEVETEMGPSVTIMKPILRFLQLLCENHNQDLQNFLRIQNNKTNYNLVSETLQFLDIMC

h_Ip3r  1916 GSTTGGLGLLGLYINEDNVGLVIQTLETLTEYCQGPCHENQTCIVTHESNGIDIITALIL
zf_Ip3r 1898 GSTTGGLGLLGLYINENNVELITQTLETLTEYCQGPCQENQTCIVSHECNGIDIITALIL

h_Ip3r  1976 NDISPLCKYRMDLVLQLKDNASKLLLALMESRHDSENAERILISLRPQELVDVIKKAYLQ
zf_Ip3r 1958 NDISPLCRYRMELVLQLKDNASKLLLALMESRHDSENAERILFNLRPRELVELIKKAYLQ

h_Ip3r  2036 EEERENSEVSPREVGHNIYILALQLSRHNKQLQHLLKPVKRIQEEEAEGISSMLSLNNKQ
zf_Ip3r 2018 EGECKEGEVSPREVGHNIYILALQLARHNKVLLTLLKPVKKIKEEEEESISSMLNLNKQQ

h_Ip3r  2096 LSQMLKSSAPAQEEEEDPLAYYENHTSQIEIVRQDRSMEQIVFPVPGICQFLTEETKHRL
zf_Ip3r 2078 E-----------EEKEDPLEHYDRQTAQIEIVREDRSMEQIVFPVHPICEFLTEESKFRV

h_Ip3r  2156 FTTTEQDEQGSKVSDFFDQSSFLHNEMEWQRKLRSMPLIYWFSRRMTLWGSISFNLAVFI
zf_Ip3r 2127 FTTTEQDEQGSKVTNFFEQTSFLHNEMEWQKKLRSMPVLYWFSRRMSLWGTISFNLAVFI

h_Ip3r  2216 NIIIAFFYPYMEGASTGVLDSPLISLLFWILICFSIAALFTKRYSIRPLIVALILRSIYY
zf_Ip3r 2187 NLIIALFYPHDSGHS-GSIDSSLLLMGFWCFAGLAVLGLLFKRYGFQSLTVAITLRCIYH

h_Ip3r  2276 LGIGPTLNILGALNLTNKIVFVVSFVGNRGTFIRGYKAMVMDMEFLYHVGYILTSVLGLF
zf_Ip3r 2246 FGIGPTLLLLGALNLINKIVYLVSFVGNNGTFIMGYKAMVMDVEFLYHVAYVLTSSLGLF

h_Ip3r  2336 AHELFYSILLFDLIYREETLFNVIKSVTRNGRSILLTALLALILVYLFSIVGFLFLKDDF
zf_Ip3r 2306 VHEFFYSILLFDLIYREETLFNVIKSVTRNGRSILLTAVLAIILVYLFSIVGFLFLRNDF

h_Ip3r  2396 ILEVDRLPNNHSTASPLGMPHGAAAFVDTCSGDKMDCVSGLSVPEVLEEDRELDSTERAC
zf_Ip3r 2366 IMEVDHLAS--------PAPGDTESFMSSCSSDGIDCTEETGLLAPAEEDE--DNTERAC

h_Ip3r  2456 DTLLMCIVTVMNHGLRNGGGVGDILRKPSKDESLFPARVVYDLLFFFIVIIIVLNLIFGV
zf_Ip3r 2416 DTLLMCIITVLNHGLRNGGGVGDVLRKPSKNEPLFPARVVYDLLFYFIVIIIVLNLIFGV
```

```
h_Ip3r  2516 IIDTFADLRSEKQKKEEILKTTCFICGLERDKFDNKTVSFEEHIKLEHNMWNYLYFIVLV
zf_Ip3r 2476 IIDTFADLRSEKQKKEEVLKTTCFICGLERDKFDNKTVSFEEHIKLEHNIWNYLYFIVLI

h_Ip3r  2576 RVKNKTDYTGPESYVAQMIKNKNLDWFPRMRAMSLVSNEGEGEQNEIRILQDKLNSTMKL
zf_Ip3r 2536 REKNKTDYTGPESYVALMIKNKNLDWFPRMQAMSLVVTDGDGEQNEMRNLQDRLSSTMKV

h_Ip3r  2636 VSHLTAQLNELKEQMTEQRKRRQRLGFVDVQNCISR-----------------
zf_Ip3r 2596 VTQLTSQLTELKEQMTEQRKRRQRMGFVDVQSGSNPGMPVPPSPAGGNQVYKA
```

```
h_Pi4kb      1 ---------MRFLEARSLAVAMGDTVVEPAPLKPTSEP---TSGPPGNNGGS-----LLS
zf_Pi4kb     1 MGDTELELSPTHLEELQKSPSTSTTSSLSLPSSPSSGPHPLTSSSPSTSEGLPTSSPPLD

h_Pi4kb     44 VITEGVGELS-VIDPEVAQKACQEVLEKVKLLHGGVAVSSRGTPLELVNGDGVDSEIRCL
zf_Pi4kb    61 VISEGLGELSLVIDTEVAKKACQEVLQKVKFLKGDGEVSSASSEPILANG-TAHPEANDG

h_Pi4kb    103 DDPPAQIREEEDEMGAAVASGTAKGARRRRQNNSAKQSWLLRLFESKLFDISMAISYLYN
zf_Pi4kb   120 GQPP-KISEEEVEP--------IKSVRRRQKNNSSKQSWLLRLFESKLFDISMAISYLYN

h_Pi4kb    163 SKEPGVQAYIGNRLFCFRNEDVDFYLPQLLNMYIHMDEDVGDAIKPYIVHRCRQSINFSL
zf_Pi4kb   171 SKEPGVQAYIGNRLFSFRNEDVDFYLPQLLNMYIHMDEDVGDAIKPYVVYRCRQSINFSL

h_Pi4kb    223 QCALLLGAYSSDMHISTQRHSRGTKLRKLILSDELKPAHR--KRELPSLSP---------
zf_Pi4kb   231 QCAWLLGAYSSDMHISTQRHSRGTKLRKLILSDELKPSSQRIRREVPQPPPPYPPPLHHG

h_Pi4kb    272 --APDTGLSPSKRTHQRSKSDATASISLSSNLKRTASNPKVENEDEELSSSTESIDNSFS
zf_Pi4kb   291 PGMSEHSLSPSKRTHQRSKSDATVSISLSSNLKRTASNPKVETSQDELSFPLS--TSFLS

h_Pi4kb    330 SPVRLAPEREFIKSLMAIGKRLATLPTKEQKTQRLISELSLLNHKLPARVWLPTAGFDHH
zf_Pi4kb   349 QPVRLTPQREFIKSLMGIGKRLATLPTKEQKTQRLISELSLLNHKLPARVWLPTAAFDHH

h_Pi4kb    390 VVRVPHTQAVVLNSKDKAPYLIYVEVLECENFDTTSVPARIPENRIRSTRSVENLPECGI
zf_Pi4kb   409 VVRVPHTQAVVLNSKDKAPYLIYVEVLECENFETSSVPVRIPETQIRSTRSVENLPDCGI

h_Pi4kb    450 THEQRAGSFSTVPNYDNDDEAWSVDDIGELQVELPEVHTNSCDNISQFSVDSITSQESKE
zf_Pi4kb   469 TPDQRASSFSTVPNYDNDDEAWSVDDIGELQVELPEIHTNSCDNISQFSVDSITSQESKE

h_Pi4kb    510 PVFIAAGDIRRRLSEQLAHTPTAFKRDPEDPSAVALKEPWQEKVRRIREGSPYGHLPNWR
zf_Pi4kb   529 PIFIAAGDIRRRLSEQLAHTPTTFRKDPEDPSAVALKEPWQEKVRRIREGSPYGHLPNWR

h_Pi4kb    570 LLSVIVKCGDDLRQELLAFQVLKQLQSIWEQERVPLWIKPYKILVISADSGMIEPVVNAV
zf_Pi4kb   589 LLSVIVKCGDDLRQELLAYQVLKQLQIIWEQERVPLWIKPYKILVISSDSGMIEPVVNAV

h_Pi4kb    630 SIHQVKKQSQLSLLDYFLQEHGSYTTEAFLSAQRNFVQSCAGYCLVCYLLQVKDRHNGNI
zf_Pi4kb   649 SIHQVKKQSQLLLLDYFRQEHGNFNTEEFLTAQRNFVQSCAGYCLICYLLQVKDRHNGNI

h_Pi4kb    690 LLDAEGHIIHIDFGFILSSSPRNLGFETSAFKLTTEFVDVMGGLDGDMFNYYKMLMLQGL
zf_Pi4kb   709 LLDSEGHIIHIDFGFILSSSPRNLGFETSAFKLTSEFVDVMGGLDGDMFNYYKMLMLQGL

h_Pi4kb    750 IAARKHMDKVVQIVEIMQQGSQLPCFHGSSTIRNLKERFHMSMTEEQLQLLVEQMVDGSM
zf_Pi4kb   769 IAARKHMEKVIQIVEIMQQGSQLPCFHGSSTIRNLKERFHMNLTEEQLQVLVEQMVDGSM
```

```
h_Pi4kb   810 RSITTKLYDGFQYLTNGIM-----------------------------------------
zf_Pi4kb  829 RSITTKLYDGFQYLTNGIMDEDSLILGHLLVVAKNIAKKEGLAEGYRVVINDGKNGAQSV

h_Pi4kb       ------------------
zf_Pi4kb  889 YHLHIHVLGGRQMKWPPG
```

```
h_Pink1      1 MAVRQALGRGLQLGRALLLRFTGKPGRAYGLGRPGPAAGCVRGERPGWAAGPGAEPRRVG
zf_Pink1     1 MSVKHVLSRGLELGRSVFQLGLLKP---------AGRVAAKFRGER--LRVSRPTRTVQPQ

h_Pink1     61 LGLPNRLRFFRQSVAGLAARLQRQFVVRAWGCAGPCGRAVFLAFGLGLGLIEEKQAESRR
zf_Pink1    51 TFLPGRYRFFRLSVSGLAAQLQSGAFRRVIGGGSARNRAVFLAFGVGLGLIEQEQEEDRT

h_Pink1    121 AVSACQEIQAIFTQK-SKPGPDPLDTRRLQGFRLEEYLIGQSIGKGCSAAVYEATMPTLP
zf_Pink1   111 SAALCQEIQAVFRKKKFQSLPKPFTS----GYRLEDYVIGKQIGKGCNAAVYEAAAPFAP

h_Pink1    180 QNLEVTKSTGLLPGRGPGTSAPGEGQERAPGAPAFPLAIKMMWNISAGSSSEAILNTMSQ
zf_Pink1   167 PVESKKCSLVELNPKEAEDDNKKEEPLRLSASPSFPLAMKMMWNIGAGSSSDAILRSMSM

h_Pink1    240 ELVPASRVALAGEYGAVTYR-KSKRGPKQLAPHPNIIRVLRAFTSSVPLLPGALVDYPDV
zf_Pink1   227 ELVPSCPQALRKEQGELTLNGHFGAVPKRLSAHPNVITVYRAFTAEVPLLPGAREEYPDV

h_Pink1    299 LPSRLHPEGLGHGRTLFLVMKNYPCTLRQYLCVNTPSPRLAAMMLLQLLEGVDHLVQQGI
zf_Pink1   287 LPARLNPHGLGSNRTLFLVMKNYPCTLRQYLEVCVPKRTQASLMFLQLLEGVDHLCRQNI

h_Pink1    359 AHRDLKSDNILVELDPDGCPWLVIADFGCCLADESIGLQLPFSSWYVDRGGNGCLMAPEV
zf_Pink1   347 AHRDLKSDNILLEFDNTGCPRLVITDFGCCLAEDS-GLKLPFSSWWVNRGGNSCLMAPEV

h_Pink1    419 STARPGPRAVIDYSKADAWAVGAIAYEIFGLVNPFYGQGKAHLESRSYQEAQLPALPESV
zf_Pink1   406 STAVPGPGVVIDYSKADVWAVGAIAYELFGQPNPFY-----TLESRSYQEKQLPALPAAA

h_Pink1    479 PPDVRQLVRALLQREASKRPSARVAANVLHLSLWGEHILA-LKNLKLDKMVGWLLQQSAA
zf_Pink1   461 PDDVQLVVKLLLRKNPHKRPSARVAANILHISLWGRRVLAGLDKVQMAEMMAWLQCQSAV

h_Pink1    538 TLLANRLTEKCCVETKMKMLFLANLECETLCQAALLLCS----WRAAL------
zf_Pink1   521 VLLKGRGRDQSSVEAELQRSFLANIELEDLRTAVSFLTYERKQWRYLLMSNSQP
```

```
h_Slc25a25      1 MLCLCLYVPVIGEAQTEFQYFESKGLPAELKSIFKLSVFIPSQEFSTYRQWKQKIVQAGD
zf_Slc25a25     1 MLCLCLYVPVHNSDQIEVEYFESNGLPSELKSLKSLSVLLPSQEFSTYRRWRKKSLKTEE

h_Slc25a25     61 KDLDGQLDFEEFVHYLQDHEKKLRLVFKSLDKKNDGRIDAQEIMQSLRDLGVKISEQQAE
zf_Slc25a25    61 KEHDGQLDFEEFVHYLQDHEKDLKLVFKSMDRKIAGQVNANDIVNSLRDLGVHISLKQAE

h_Slc25a25    121 KILKSMDKNGTMTIDWNEWRDYHLLHPVENIPEIILYWKHSTIFDVGENLTVPDEFTVEE
zf_Slc25a25   121 KVLKSMDKNGTMTIDWNEWKKYPTLQPAENIPEIILYWKHSTIFDVGESLMVPDEFTVEE

h_Slc25a25    181 RQTGMWWRHLVAGGGAGAVSRTCTAPLDRLKVLMQVHASRSNNMGIVGGFTQMIREGGAR
zf_Slc25a25   181 HLTGMWWRHLVSGGGAGAVSRTCTAPLDRLKVLMQVHGCQGKSMCLMSGLTQMIKEGGVR

h_Slc25a25    241 SLWRGNGINVLKIAPESAIKFMAYEQIKRLVGSDQETLRIHERLVAGSLAGAIAQSSIYP
zf_Slc25a25   241 SLWRGNGINVIKIAPETALKFMAYEQIKRVMGSSQETLGISERFVAGSLAGVIAQSTIYP

h_Slc25a25    301 MEVLKTRMALRKTGQYSGMLDCARRILAREGVAAFYKGYVPNMLGIIPYAGIDLAVYETL
zf_Slc25a25   301 MEVLKTRLALRKTGQYKGISDCAKHILKTEGMSAFYKGYVPNMLGIIPYAGIDLAVYETL

h_Slc25a25    361 KNAWLQHYAVNSADPGVFVLLACGTMSSTCGQLASYPLALVRTRMQAQASIEGAPEVTMS
zf_Slc25a25   361 KNTWLQRYGTENADPGVFVLLACGTVSSTCGQLASYPLALIRTRMQAQASVEGSSQVSMT

h_Slc25a25    421 SLFKHILRTEGAFGLYRGLAPNFMKVIPAVSISYVVYENLKITLGVQSR
zf_Slc25a25   421 GLFKQIMKTEGPTGLYRGLTPNFLKVIPAVSISYVVYEHIKSTLGVRSR
```

```
h_Trpc1      1 ------------------------------------------------------------
zf_Trpc1     1 MGISEEEWERLQKALEWPSPDQEITELSESTSPDHSTFTIVGLNQNYSVGEKILVTITSR

h_Trpc1      1 ------------------------------------------------------------
zf_Trpc1    61 DHNNKLKRYGGDFFKVKLFNSELKASVYGEVVDHRNGTYSASLLLPWEGQAQVSVRLEHS

h_Trpc1      1 ------------------------------------------------------------
zf_Trpc1   121 SEVVQILKKYRESSFTRSHYNGHFEGPGPNKTRISEVVPCNLKWGGNGSWIKGNCCCEYK

h_Trpc1      1 ------------------------------------------------------------
zf_Trpc1   181 DIKTGTVWHCIKRMDLHTPPTGGPLMAVELTNNIIVHWGPHGVPLRFTKMLITDLHYISN

h_Trpc1      1 ------------------------------------------------------------
zf_Trpc1   241 DIDEIAGGSHAVIVFTIAAHLVFHPLTFYVHEVAKIRQSVVSLLSRAPETLVIIKSGNTA

h_Trpc1      1 -----------------MMAALYPSTD---------------------------LSGASS
zf_Trpc1   301 GQKDIFQSDWYAMQLNTVMQEMFRDIDAVIYFDVWQMTSCHYLHDDVHPGCKALLSGMHI

h_Trpc1     17 SSLPSSP-----------SSSSPNEVMALKDVREVKEENTLNEKLFLLACDKGDYYMVKK
zf_Trpc1   361 SALPYSLSVIMAALYQGTDSSSPDKYLALKDVREVKEETTLDEKLFLLACEKGDYYMVKK

h_Trpc1     66 ILEENSSGDLNINCVDVLGRNAVTITIENENLDILQLLLDYGCQKLMERIQNPEYSTTMD
zf_Trpc1   421 LLEEKRHGELNINCVDVLGRDAVTISIENENLDILQLLLDHGCQKLIQKIQNPEYSTTMD

h_Trpc1    126 VAPVILAAHRNNYEILTMLLKQDVSLPKPHAVGCECTLCSAKNKKDSLRHSRFRLDIYRC
zf_Trpc1   481 VAPVILAAHRNNYEILTMLLKQDISLPRPHAVGCECTLCNAKNKKDSLRHSRFRLDIYRC

h_Trpc1    186 LASPALIMLTEEDPILRAFELSADLKELSLVEVEFRNDYEELARQCKMFAKDLLAQARNS
zf_Trpc1   541 LASPSLIMLTEEDPILRAFELSADLKELSLVEVEFRNDYEELAKQCKMFAKDLLAQARNS

h_Trpc1    246 RELEVILNHTSSDEPLDKRGLLEERMNLSRLKLAIKYNQKEFVSQSNCQQFLNTVWFGQM
zf_Trpc1   601 RELEVILNHTSSEDHVDKRGLLEERMNLSRLKLAIKYNQKEFVAQSNCQQFLNTVWFGEM

h_Trpc1    306 SGYRRKPTCKKIMTVLTVGIFWPVLSLCYLIAPKSQFGRIIHTPFMKFIIHGASYFTFLL
zf_Trpc1   661 ASYRRKHTCLKIVSVLSVALLWPLLSICYLLGPRSRVGQVIHTPFIKFIIHSASYFTFLL

h_Trpc1    366 LLNLYSLVYNEDKKNTMGPALERIDYLLILWIIGMIWSDIKRLWYEGLEDFLEESRNQLS
zf_Trpc1   721 LLNLYSLIYN-DKKN-MAPALQFIDYLLILWIIGMVWSDVKRLWYEGLEDFLEESRNQLS

h_Trpc1    426 FVMNSLYLATFALKVVAHNKFHDFAD---RKDWDAFHPTLVAEGLFAFANVLSYLRLFFM
zf_Trpc1   779 FVMNSLYLATFALKIVAHNKYSKIIKPEERKEWDAFHPTLVAEGLFAFAIVLSYLRLFFM
```

```
h_Trpc1    483 YTTSSILGPLQISMGQMLQDFGKFLGMFLLVLFSFTIGLTQLYDKG----YTSKEQKDCV
zf_Trpc1   839 YTTSSILGPLQISMGQMLQEFGKFLGLFLLVLISFTIGLTQLYGKDQDPSKTKDDNKDCE

h_Trpc1    539 GIFCEQQSNDTFHSFIGTCFALFWYIFSLAHVAIFVTRFSYGEELQSFVGAVIVGTYNVV
zf_Trpc1   899 GIYCQQQSNDTFHTFMGTCYALFWYIFSLAHVNLFVTRISYTEELRSFVGALIVGTYNIV

h_Trpc1    599 VVIVLTKLLVAMLHKSFQLIANHEDKEWKFARAKLWLSYFDDKCTLPPPFNIIPSPKTIC
zf_Trpc1   959 VVIVLTKLLVAMLHKSFRQIANHEDKEWKFARAKLWLSYFDDKCTLPPPFNVLPSPKTVC

h_Trpc1    659 YMISSLSKWICSHTSKGKVKRQNSLKEWRNLKQKRDENYQKVMCCLVHRYLTSMRQKMQS
zf_Trpc1  1019 YLVISMSKWICSHTSTGKVKRQNSLREWTNLKQKRDQNYQKIMCCLVHRYLTSTRQKMQS

h_Trpc1    719 TDQATVENLNELRQDLSKFRNEIRDLLGFRTSKYAMFYPRN
zf_Trpc1  1079 MDQATVENLNDLRQDLSKFRNEMRDLLGFRTSKYAMFYPRS
```

```
h_Trpc5      1 ---MAQLYYKKVNYSPYRDRIPLQIVRAETELSAEEKAFLNAVEKGDYATVKQALQEAEI
zf_Trpc5     1 MNPMSHLYYKKSSYSPYRDRIPLQIVRAEVELSPEERAFLSAVEKGDYAGVQHALREAEV

h_Trpc5     58 YYNVNINCMDPLGRSALLIAIENENLEIMELLLNHSVYVGDALLYAIRKEVVGAVELLLS
zf_Trpc5    61 YYNIDTNCVDPLGRSALLIAIENENLEVMELLLDHGVNTGDALLYAIRKEVVGAVELLLS

h_Trpc5    118 YRRPSGEKQVPTLMMDTQFSEFTPDITPIMLAAHTNNYEIIKLLVQKRVTIPRPHQIRCN
zf_Trpc5   121 HRKPSGEKQVPSLMMDAQFSEFTPDITPIMLAAHTNNYEIIKLLVQRKVTIPRPHQIRCD

h_Trpc5    178 CVECVSSSEVDSLRHSRSRLNIYKALASPSLIALSSEDPILTAFRLGWELKELSKVENEF
zf_Trpc5   181 CVECVSSSEVDSLRHSRSRLNIYKALASPSLIALSSEDPILTAFRLGWELKELSKVENEF

h_Trpc5    238 KAEYEELSQQCKLFAKDLLDQARSSRELEIILNHRDDHSEELDPQKYHDLAKLKVAIKYH
zf_Trpc5   241 RQEYEELSQQCKLFAKDLLDQARSSRELETILNHRDDQSEELDPRECRDLAKLKLAIKYH

h_Trpc5    298 QKEFVAQPNCQQLLATLWYDGFPGWRRKHWVVKLLTCMTIGFLFPMLSIAYLISPRSNLG
zf_Trpc5   301 QKEFVAQPNCQQLLATLWYDGFPGWRRRHWAVKLVMCFIIGLLFPVFSLVYLIAPKSTLG

h_Trpc5    358 LFIKKPFIKFICHTASYLTFLFMLLLASQHIVRTDLHVQGPPPTVVEWMILPWVLGFIWG
zf_Trpc5   361 LFIKKPFIKFICHTASYLTFLFLLLLASQHIARTNLHMQGPPPTVVEWMILPWVLGFIWA

h_Trpc5    418 EIKEMWDGGFTEYIHDWWNLMDFAMNSLYLATISLKIVAYVKYNGSRPREEWEMWHPTLI
zf_Trpc5   421 EIKEMWDGGFNEYVHDWWNLMDFAMNSLYLATISLKIVAYVKYNSSRPREEWEMWHPTLI

h_Trpc5    478 AEALFAISNILSSLRLISLFTANSHLGPLQISLGRMLLDILKFLFIYCLVLLAFANGLNQ
zf_Trpc5   481 AEALFAIANIFSSLRLISLFTANSHLGPLQISLGRMLLDILKFLFIYCLVLLAFANGLNQ

h_Trpc5    538 LYFYYETRAIDEPNNCKGIRCEKQNNAFSTLFETLQSLFWSVFGLLNLYVTNVKARHEFT
zf_Trpc5   541 LYFYYETEAADEPNHCKGIRCERQNNAFST------------------------------

h_Trpc5    598 EFVGATMFGTYNVISLVVLLNMLIAMMNNSYQLIADHADIEWKFARTKLWMSYFDEGGTL
zf_Trpc5       ------------------------------------------------------------

h_Trpc5    658 PPPFNIIPSPKSFLYLGNWFNNTFCPKRDPDGRRRRRNLRSFTERNADSLIQNQHYQEVI
zf_Trpc5       ------------------------------------------------------------

h_Trpc5    718 RNLVKRYVAAMIRNSKTHEGLTEENFKELKQDISSFRYEVLDLLGNRKHPRSFSTSSTEL
zf_Trpc5       ------------------------------------------------------------

h_Trpc5    778 SQRDDNNDGSGGARAKSKSVSFNLGCKKKTCHGPPLIRTMPRSSGAQGKSKAESSSKRSF
zf_Trpc5       ------------------------------------------------------------
```

```
h_Trpc5   838 MGPSLKKLGLLFSKFNGHMSEPSSEPMYTISDGIVQQHCMWQDIRYSQMEKGKAEACSQS
zf_Trpc5      ------------------------------------------------------------

h_Trpc5   898 EINLSEVELGEVQGAAQSSECPLACSSSLHCASSICSSNSKLLDSSEDVFETWGEACDLL
zf_Trpc5      ------------------------------------------------------------

h_Trpc5   958 MHKWGDGQEEQVTTRL
zf_Trpc5      ----------------
```

```
h_Vamp2      1 MSATAATAPPAAPAGEGGPPAPPPNLTSNRRLQQTQAQVDEVVDIMRVNVDKVLERDQKL
zf_Vamp2     1 -----MSAPAGAPAPEGGNQAPP-NLTSNRRLQQTQAQVDEVVDIMRVNVDKVLERDQKL

h_Vamp2     61 SELDDRADALQAGASQFETSAAKLKRKYWWKNLKMMIILGVICAIILIIIIVYFST
zf_Vamp2    55 SELDDRADALQAGASQFETSAAKLKNKYWWKNAKMMIILGVICVIVLIIIIVYFST
```
