# Supplementary material for: Proteomic and functional analysis of NCS-1 binding proteins reveals novel signaling pathways required for inner ear development in zebrafish
Source: BMC Neurosci. 2009 Mar 25;10:27. doi: 10.1186/1471-2202-10-27 (PMC2679751; doi:10.1186/1471-2202-10-27)
Supplement: Additional File 2 — Expression of zebrafish NBPs in head region. Whole mount in situ hybridization analysis was performed at 24, 48, and 72 hpf. Expression profiles in head region are shown for all of the NBPs represented in Figure 1. Genes are grouped according to presumed functional properties. All images are lateral views of the head, anterior to the left. [file 1471-2202-10-27-S2.pdf]

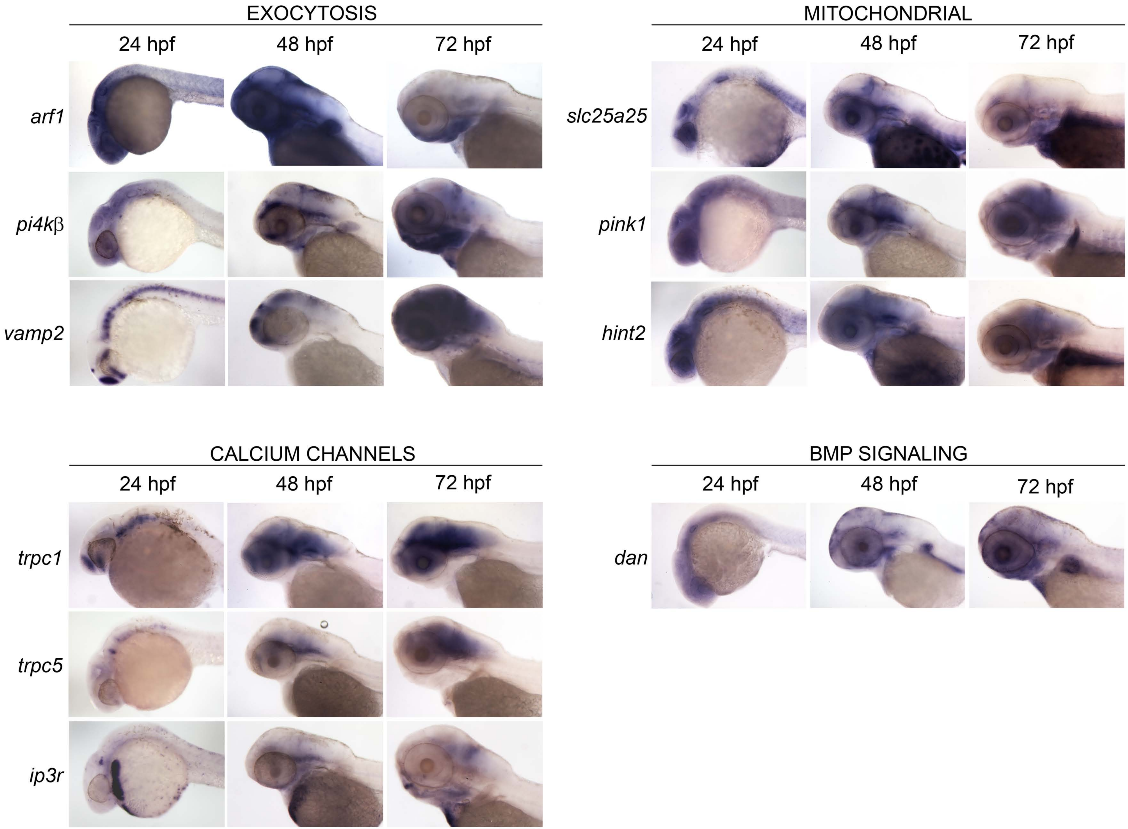
EXOCYTOSIS
24 hpf
48 hpf
72 hpf
arf1
pi4kβ
vamp2
MITOCHONDRIAL
24 hpf
48 hpf
72 hpf
slc25a25
pink1
hint2
CALCIUM CHANNELS
24 hpf
48 hpf
72 hpf
trpc1
trpc5
ip3r
BMP SIGNALING
24 hpf
48 hpf
72 hpf
dan
